# The impact of rainfall on the temporal and spatial distribution of taxi passengers

**DOI:** 10.1371/journal.pone.0183574

**Published:** 2017-09-05

**Authors:** Dandan Chen, Yong Zhang, Liangpeng Gao, Nana Geng, Xuefeng Li

**Affiliations:** School of Transportation, Southeast University, Nanjing, Jiangsu Province, China; Beihang University, CHINA

## Abstract

This paper focuses on the impact of rainfall on the temporal and spatial distribution of taxi passengers. The main objective is to provide guidance for taxi scheduling on rainy days. To this end, we take the occupied and empty states of taxis as units of analysis. By matching a taxi's GPS data to its taximeter data, we can obtain the taxi's operational time and the taxi driver's income from every unit of analysis. The ratio of taxi operation time to taxi drivers' income is used to measure the quality of taxi passengers. The research results show that the spatio-temporal evolution of urban taxi service demand differs based on rainfall conditions and hours of operation. During non-rush hours, taxi demand in peripheral areas is significantly reduced under increasing precipitation conditions, whereas during rush hours, the demand for highly profitable taxi services steadily increases. Thus, as an intelligent response for taxi operations and dispatching, taxi services should guide cruising taxis to high-demand regions to increase their service time and ride opportunities.

## Introduction

Previous studies have shown that weather can significantly influence transportation demand[1], with rainfall being the strongest factor among weather parameters that influence road traffic volume [2]. For example, in recent years, hailing a taxi on rainy days has become a common problem for residents of large cities and is a source of great inconvenience to urban residents [3]. Based on a statistical analysis of taximeters, one study found that on rainy days, New York City taxi drivers earn more money than usual for each trip; therefore, by meeting their income targets in advance, taxi drivers can reduce their working hours [4]. However, another study suggested that the traffic congestion resulting from rainy weather has a negative impact on taxi drivers' work satisfaction. Other than making money, taxi drivers have no motivation to maintain specific working hours [5]. However, both these studies were performed from an economic perspective, and the study areas were in New York City. Chinese people live in different cultural and economic conditions; consequently, the conclusions of the existing studies may not apply to the behaviour of Chinese taxi drivers in rainy weather. Therefore, it is necessary to investigate Chinese taxi operations on rainy days to provide taxi scheduling guidance for China.

The most promising method to improve the efficiency of Chinese taxi drivers is for them to acquire passengers quickly and frequently[6]. Consequently, obtaining the spatial and temporal distribution characteristics of the demand for taxi services has vital practical significance in helping traditional taxi operators improve their operational efficiency[7]. Fortunately, global positioning system (GPS) technology, along with the development of satellite sensor and data communication, has gradually expanded from a solely military function to one usable by civilians [8]. With the encouragement of transportation departments and all levels of government, almost all Chinese taxis are now equipped with GPS receivers. When coupled with other technology, GPS devices installed in taxis can regularly send vehicle information such as location coordinates, direction, speed and passenger status to the taxi dispatch centre [9]. Based on these abilities, using available technologies and information, the spatial and temporal distribution characteristics of taxi service demand can be comprehensively acquired[10].

This paper proposes a standard for taxi passengers that takes both the demand of taxi passengers and taxi drivers' incomes into account. Using taxi GPS data and taximeter data, we investigated the differences between the spatial and temporal distribution characteristics of taxi service demand on sunny days and rainy days. The remainder of this paper is organized as follows. In Section 2, we review existing studies on taxi services and the effects of weather on transportation. Section 3 describes the data used for this study and the data processing flow. Section 4 introduces the definition and the quantitative methods used to investigate the provided concept, while in Section 5, we analyse the differences in characteristics between sunny and rainy days. Finally, Section 6 concludes the paper and suggests directions for future research.

## Literature review

In this paper, the literature review is presented in two sections. We first sum up the research on taxi operations that involve data mining. Then, we introduce related studies concerning the effects of weather on transportation.

### Related studies on urban taxi services

With the development of geographical information technology, using GPS devices installed in taxis to collect taxi operation traces and study their service strategies has become a research hotspot. Using taxi GPS data, some scholars performed a series of taxi service predictions to improve the operational efficiency of taxi drivers. For example, by analysing the number of taxi passengers picked up at a given time and location, Zhang et al.[6] andLi et al.[11]were able to forecast the passenger wait time and the probability of hailing a vacant taxi for city residents. Li et al.[12] and Zhang et al.[13] proposed a spatio-temporal visualization analysis method to quantify the characteristic parameters of taxi operations. Hu et al.[14], Liu et al.[15] and Cai et al. [16] investigated the differences between taxi service modes and income among various taxi drivers. Takayama et al.[7] and Chang et al.[17] predicted the demand distribution and the best queuing locations for taxi drivers. Using data-mining technology, some scholars have focused on the relationships between pick-up and drop-off locations to discuss the global mechanisms of taxi service [10, 18–20]. Additionally, based on taxi trajectory data, Ma et al.[21]proposed a convolutional neural network-based method to study the spatial-temporal evaluation of traffic flow and achieved good effect compared with three other deep learning architectures. A mapping-to-cells method proposed by He et al.[22] was used to identify freeway network bottlenecks and predict traffic congestion accurately without installing large-scale loop detectors. Kong et al[23] proposed a time-location-relationship (TLR) model to predict passenger distribution for different social functional regions. Qin et al [24] applied a multilevel ordered logit model to investigate the significant factors that influence taxi driver’s incomes using data from 8,000 taxis in Shanghai. Yamamoto et al. [25, 26]developed an adaptive routing method to help taxi drivers acquire passengers more effectively.

In general, studies on taxi services that employ taxi GPS data have focused primarily on taxi drivers. Studying taxi drivers' behaviour to discover better taxi service strategies has become a mainstream research focus. However, relatively few papers have focused exclusively on the more general behaviours of taxi services or on the spatio-temporal distribution of taxi passengers. As major participants in a taxi transaction, understanding taxi passengers' demands is important to help taxi drivers reduce their cruising times and, thus, improve their operational efficiency. Therefore, it is imperative to analyse passenger patterns over the duration of entire taxi services.

### Studies of the effects of weather on transportation

Weather conditions are considered exogenous factors that can significantly affect transportation from such aspects as travel demand, traffic flow, individual travel patterns and so on. Therefore, research topics investigating the relationships between weather factors, travel behaviour and transit passengers have attracted numerous scholars. Among these studies, travel demand, travel safety and travel behaviours have become the three central branches in research that considers the influences of weather. For instance, Koetse and Rietveld found that changes in weather conditions affect the competitive positions of various transportation modes[27]; Arana et al. analysed the impact of weather conditions on the number of public buses[28], and Cravo et al. found that bad weather conditions can have a negative effect on transit passenger flow [29]. Bocker, Cools and Creemers performed comprehensive investigations into the impacts of various weather conditions on travel behaviour [30–32]. From the aspect of transit safety, Theofilatos and Yannis conducted a literature review, while Bijleveld and Churchill reported on the effects of weather using a variety of methods [33, 34].

From the preceding literature, studies on the impact of weather have focused most heavily on mass transit, railways and private cars; only a few studies have focused on taxicabs. However, taxis are an indispensable part of city public transportation—and weather may cause disruptions to both service processing and to the temporal and spatial distribution of taxi passengers. Considering the impact of weather is necessary to improve taxi service operational efficiency. Therefore, the main goal of this paper is to explore how rainfall (which has been found to be the most important factor among all weather conditions) influences taxi services and taxi passengers [2]. The detailed methods and processes used to investigate this topic are described in more detail in the next section.

## Data sources and data processing

This study focused on a medium-sized city in China. A large-scale, real-world taxi GPS dataset comprising a period of five months was obtained for two large cities in China (Wuxi and Kunming). Wuxi and Kunming each have a population of more than 6 million people. Both cities are in a subtropical monsoon climate zone and have similar annual rainfall levels. The data for the two cities are shown in Table 1.

**Table 1:** Statistical indicators of Kunming and Wuxi.

	Area (Square Kilometers)	Population (Millions)
Kunming	2,630.79	667.7
Wuxi	1,643.88	651.1
	Annual Rainfall (Millimeters)	Motor Vehicle Ownership (Millions)
Kunming	1,035	225.5
Wuxi	1,121.7	160

In China, Kunming and Wuxi are second-tier cities; however, Kunming is the provincial capital of Yunnan Province and a famous tourist city in southwestern China, whereas Wuxi is a prefecture-level city of Jiangsu Province and a famous industrial city in southeastern China. Overall, although taxi operations occur under similar population and weather conditions in Wuxi and Kunming, the cities have different economic environments. Therefore, we selected Wuxi and Kunming to study the influence of rainy days on taxi passengers.

### Description of GPS data

In this study, we acquired a large-scale real-world dataset consisting of GPS traces of 3,500 to 4,000 taxi records per day from Wuxi and 6,000 to 7,000 taxi records per day from Kunming. The dataset covered the period from May 1st, 2015 to June 30th, 2015 in Wuxi and March 1st, 2015 to May 31st, 2015 in Kunming. The large populations and massive passenger demand in these cities both raises great challenges and provides great opportunities for taxi drivers. In our taxi GPS dataset, 8 different real-time information features were recorded at a sampling frequency of 3~12 times per minute. These features can be described as follows:

1. Record No: A unique ID number for the GPS trajectory records in the dataset;
2. Vehicle ID: A unique ID for each taxi;
3. Timestamp: A sampling time stamp recorded as the number of elapsed milliseconds since midnight on January 1, 1970;
4. Longitude: The current longitude of the encrypted World Geodetic System—1984 Coordinate System (GCJ-02 Coordinate System);
5. Latitude: The current latitude of the GCJ-02 Coordinate System;
6. Velocity: The current speed of the taxi;
7. Heading Direction: The compass direction when the current GPS trace points were recorded;
8. Running State: The current status (occupied/empty) of the taxi.

The dataset contained more than 960 million records; therefore, to reduce the statistical burden, we preprocessed the data as described in the following subsection.

### Data preprocessing and matching

**Fig 1:**
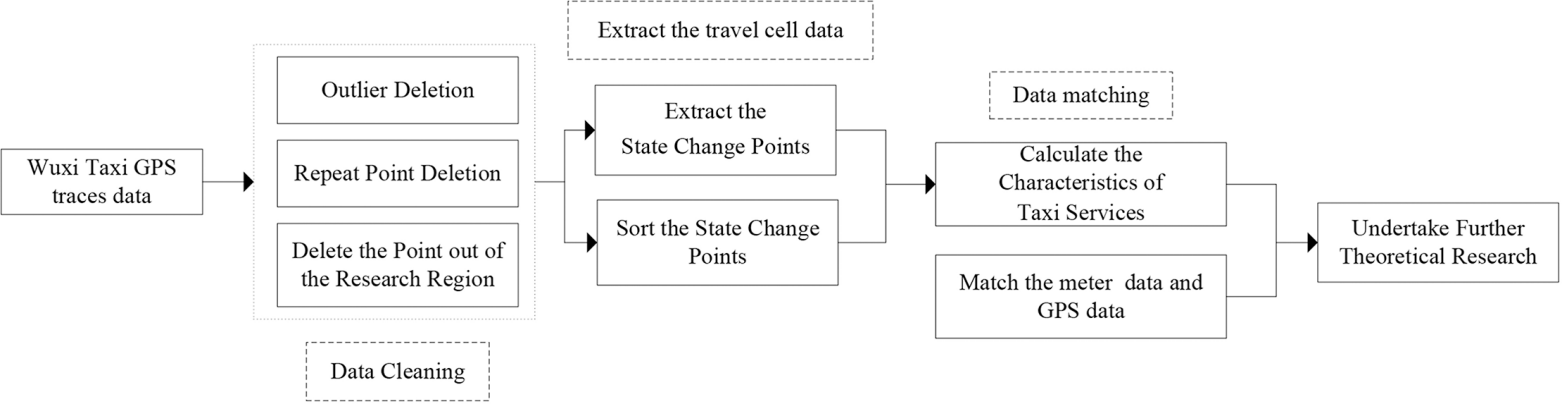
The preprocessing steps to clean and match the data.

Data quality is a longstanding issue in taxi service analysis. The movement trajectories recorded by taxi GPS devices contain the full operational behaviour of taxi drivers that can be described by some statistical variables. However, there is no need to store all the GPS data, which is huge. The preprocessing steps are shown in Fig 1.

The following list explains several of the most important steps shown in Fig 1:

(1) Extract the State Change Points

**Fig 2:**
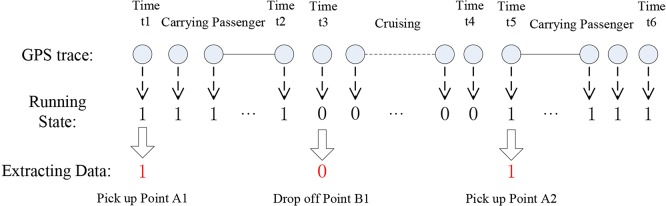
The procedure for extracting state change points.

During the data collection process, we labelled the occupied state with the number 1 and the empty (unoccupied) state with the number 0. Typically, there are several consecutive GPS trace points with the same state; consequently, we could calculate the procedure parameters by the starting points of these two adjacent states. Therefore, the extracted data was composed of the trace points in which the state number 1 and 0 alternate in one GPS trajectory record. The detailed extraction processing of these state change points is shown in Fig 2.

(2) Calculate the Characteristics of Taxi Services

Based on the result of the above analysis, we can see that in Fig 2, a trip unit includes three state change points: the first pick-up point, the drop-off point and the next pick-up point. The calculated deltas of the service time and running distance of the two adjacent points correspond to the occupied portion of the running trip and the empty portion of the running trip, respectively.

(3) Matching the Taximeter Data with State Change Points

The taximeter data contained 6 main features: the service ID of the taxi driver, the taxi license plate number, the mileage, income (fee), and the starting and ending times of the occupied running trip. For this study, we associated the income feature to the GPS trip unit when the start and end times could be closely matched to the timestamps of the first pick-up point and the drop-off point.

## Highly profitable passenger origins for taxi services

### A micro-analysis of taxi services

Taxi services are a beneficial supplement to private and public transport but have behavioural features of randomness. The study of these behavioural features is our basic starting point. From the perspective of taxi drivers, there are three basic operational modes: finding new customers, stopped and picking up customers. The three modes always occur during a taxi driver's working hours (breaks and mealtimes are not considered to be part of these modes). Therefore, in some sense, it is fair to say that taxi service is a repetitive activity that has some continuity characteristics from a spatio-temporal aspect, such as velocity, acceleration, travel routes and so on. For example, the detailed taxi service behaviours hidden in the time-stamped GPS trajectories of taxi drivers are shown in the following figure.

**Fig 3:**
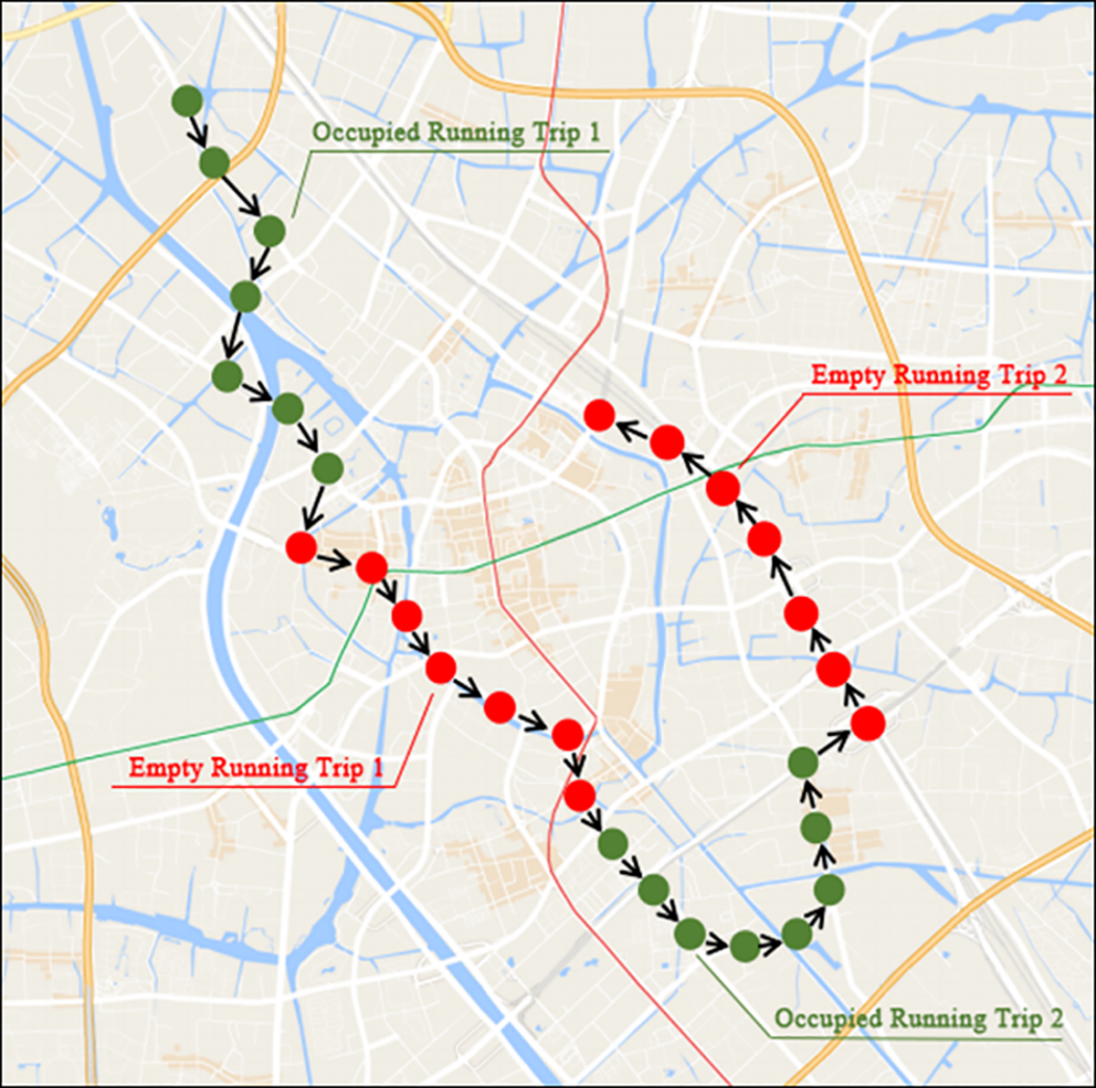
Typical GPS trajectory structures of taxi services.

As shown in Fig 3, the GPS trajectory includes both the occupied and empty running trips to describe the complete service behaviour of taxi drivers. The scope of taxi service is not limited to a specific route or area t any given time, and the occupied running trips and empty running trips tend to appear alternately and intersect at the ending point of the previous occupied running trip and the starting point of the next empty running trip. Therefore, according to these spatio-temporal features, we determined that a complete trip unit should contain both an occupied running trip and an empty running trip. A complete trip unit has the following two characteristics:

1. Each trip unit is a small segment of the full GPS trajectory of an urban taxi that completely describes a passenger-carrying activity;
2. The GPS trace points of one trip unit are recorded over some time interval, which means that a trip unit always begins with an occupied running trip and ends with an empty running trip.

### The top taxi drivers in income ranking list

**Fig 4:**
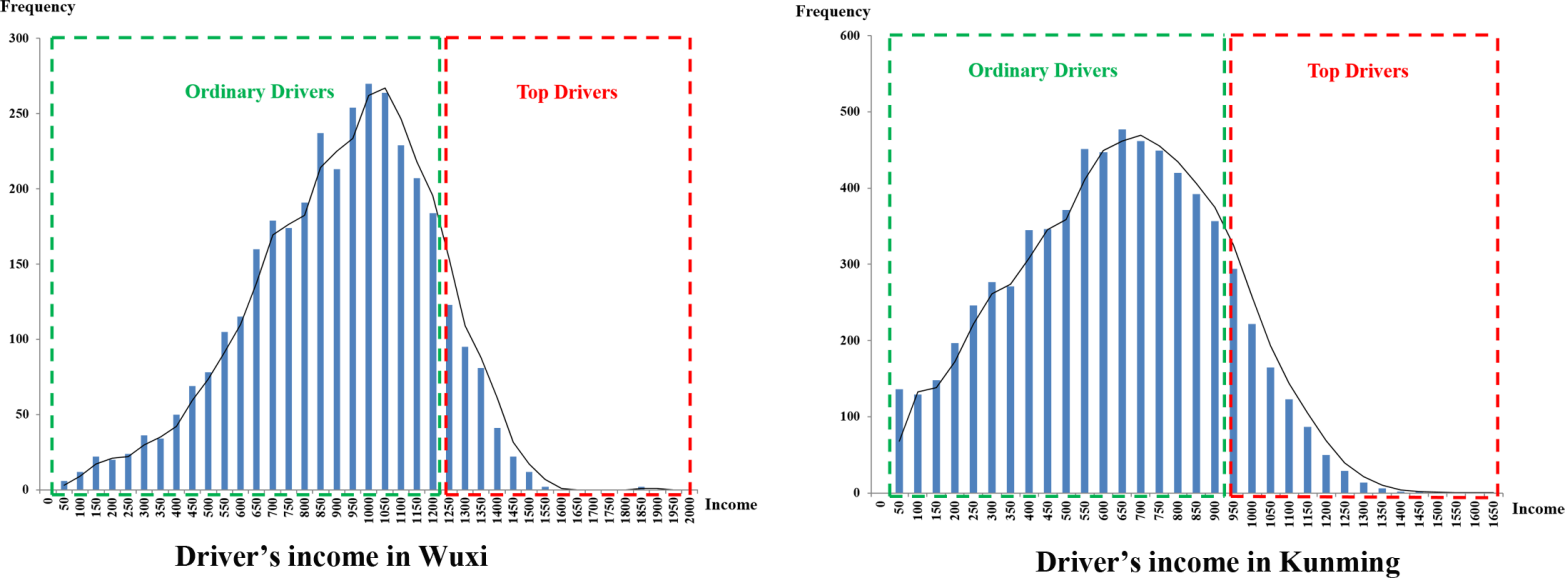
The daily income of all taxi drivers shows an approximately normal distribution.

Based on the results of previous studies[15], there is an innate connectivity between the operational efficiency and the daily income of any individual taxi. Therefore, the main purpose of this section is to perform a statistical analysis on the matched GPS trajectory and taximeter data. Fig 4 illustrates that daily income follows an approximately normal distribution for all taxi drivers over one day.

In Fig 4, the X-axis represents the daily income of taxi drivers, which ranged from 50 Yuan to 2000 Yuan, while the Y-axis represents the sample size corresponding to the daily income segments. We can see that the daily income of taxi drivers in Wuxi is higher than that of drivers in Kunming. In Wuxi, most drivers achieve an income close to the mean value (1000–1100 Yuan); however, some drivers' incomes were quite high (approximately 1250–2000 Yuan), while others had incomes that were quite low (approximately 50–400 Yuan). The relationship between daily income and the taxi drivers’ behaviours was explored by Zhang [6], who found that the average daily income follows a normal distribution for all taxi drivers. According to the division standard of taxi drivers, we classified the taxi groups in Wuxi and Kunming into high and ordinary income groups. The top drivers have an average daily income greater than 1250 Yuan in Wuxi and greater than 950 Yuan in Kunming. However, ordinary drivers have an average daily income from 800 to 1250 Yuan in Wuxi and from 500 to 950 Yuan in Kunming.

### The highly profitable passengers for taxi services

As an important urban transportation mode, taxi service is also affected by complex traffic environments. Therefore, it is only natural that the daily income of taxi drivers exhibits some volatility over an entire month. However, by comparing the characteristics of top drivers and ordinary drivers, a special phenomenon in GPS trajectory performance was revealed by the following table:

As shown in Table 2, the passenger number served by each top driver is higher than that of ordinary driver in Wuxi and Kunming. Based on the operation process, the taxi running speed of top drivers is higher than that of ordinary drivers, regardless of whether the taxi is running occupied or empty. Furthermore, the top drivers appear to spend less time and mileage on empty runs than do ordinary drivers. As a prelude to carrying passengers, cruising plays a key role in the outcome of passengers and should be combined with the empty running time when analysing taxi operations. Furthermore, the starting point of the cruse represents the end point of the last passenger. Identifying the most likely passenger origins can reduce empty running trip times and represents the best method of improving the individual operational efficiency and profits of taxi drivers. Therefore, based on the research of Sun et al. [35], in this study, we present the concept of a "highly profitable passenger" to describe passenger origins where the next passengers can be acquired quickly and, thus, improve taxi drivers' incomes. The determining formula for a highly profitable passenger in a trip unit can be expressed as follows:

$$E=\frac{I}{T}=\frac{I}{\left(T_{1}+T_{2}\right)}$$ (1)

where *E* represents the measurement rate of a highly profitable passenger, *I* is the income from carrying this passenger, and *T*_1_ and *T*_2_ refer to the time spent in the occupied running trip and the empty running trip, respectively. When the measurement rate of a passenger is ranked in the top 25%, we identify this passenger as a highly profitable passenger.

**Table 2:** Taxi operation statistics under different income levels.

	Passenger Number Served by Each Driver		Occupied Running Time (S)		Occupied Running Speed (km per hour)	
City	Wuxi	Kunming	Wuxi	Kunming	Wuxi	Kunming
Top drivers	52	46	950.32	1011.86	31.5	24.67
Ordinary drivers	35	30	860.54	1092.08	26.6	22.81
	Empty Running Time (S)			Empty Running Speed (km per hour)		
City	Wuxi		Kunming	Wuxi		Kunming
Top drivers	577.78		847.45	18.3		14.72
Ordinary drivers	897.42		1733.18	16.4		13.93

## Time and space analysis of taxi service

In this section, we investigated the differences between the temporal and spatial distributions of highly profitable passengers on sunny and rainy days. The weather data for this study were obtained from the Weather Underground website (www.wunderground.com). These data were recorded at weather stations in Wuxi and Kunming and are assumed to be representative of weather conditions across the cities. The weather data were collected at approximately 30-minute intervals and included such features as temperature, humidity, air pressure, wind speed, weather conditions and rainfall amount, all recorded as they occurred. Because this study is primarily interested in the effect of rainfall on the spatio-temporal distribution of highly profitable passengers, the humidity and rainfall amount are the features of particular concern.

### The temporal analysis of taxi service

To discuss the impact of rainfall on taxi service in more detail, we performed a statistical analysis based on the daily rainfall intensity and the daily rainfall duration. According to the precipitation intensity standards issued by China's National Meteorological Administration, the daily rainfall in Kunming and Wuxi are divided into 5 grades: heavy rain, moderate rain, showers, light rain and no rain. We analysed all the taxi trajectory data for the two cities under these five conditions and calculated the average values of different operation indices as shown in Table 3.

**Table 3:** Taxi operation statistics under different rainfall levels.

	Total Passengers		Occupied Running Time (S)		Occupied Running Speed (km per hour)	
	Wuxi	Kunming	Wuxi	Kunming	Wuxi	Kunming
Heavy Rain	125,351	208,335	769.79	1,057.77	24.96	21.69
Moderate rain	127,505	224,542	731.72	1,038.65	26.30	21.76
Shower	133,830	223,995	704.75	1,023.07	28.50	22.00
Drizzle	134,864	229,720	646.22	981.44	30.00	22.31
No rain	136,296.2	234,434.8	649.36	961.12	30.70	22.38
	Empty Running Time (S)			Empty Running Speed (km per hour)		
	Wuxi		Kunming	Wuxi		Kunming
Heavy Rain	1,425.62		1,936.53	15.98		13.88
Moderate rain	1,245.96		1,881.54	16.42		14.02
Shower	1,145.86		1,758.44	16.76		14.11
Drizzle	1,056.87		1,609.26	16.84		14.13
No rain	998.75		1,547.26	17.00		14.27

Table 3 shows that total number of passengers in Kunming is almost 1.5 times greater than that in Wuxi. As the amount of rainfall increases, the number of passengers in the two cities declined by varying degrees; greater amounts of rainfall resulted in fewer total passengers. The decline in Kunming was approximately 11.13%, greater than the decline in Wuxi. However, the results also show that the total number of passengers under moderate rain declined only slightly compared with that under showers. Therefore, we removed the moderate rain and drizzle conditions and contrasted the heavy rain, showers and no rain conditions in our spatial analysis of taxi services.

Considering China's morning and evening peak traffic congestion, the service time period was divided into rush hours and non-rush hours. Compared with the morning peak, the evening peak was mainly associated with commuting and diverse reasons for travel, and evening traffic congestion was worse. Therefore, we selected the evening peak as the research period and compared it with the conditions observed in the off-peak hours. We set the rush hour period in Kunming and Wuxi between 5 and 6 pm and the off-peak hours between 9 and 10 pm. The taxi trajectory data during those two periods were calculated. Then, the average value of the taxi operation index was calculated and is shown in Table 4.

**Table 4:** Taxi operation statistics under different rainfall periods.

	Weather	Total Passengers		Income (Yuan)		Occupied Running Time (S)	
		Wuxi	Kunming	Wuxi	Kunming	Wuxi	Kunming
Evening Rush Hour	Rain	6,258	1,1850	21.75	20.95	969.86	1,251.44
	No Rain	5,652	9511	20.78	21.12	1,035.21	1,368.96
Off-Peak Hour	Rain	7,605	1,3081	19.85	18.33	896.48	1,015.62
	No Rain	8,377	1,4410	18.56	17.64	645.24	905.17
	Weather	Occupied Running Speed (km per hour)		Empty Running Time (S)		Empty Running Speed (km per hour)	
		Wuxi	Kunming	Wuxi	Kunming	Wuxi	Kunming
Evening Rush Hour	Rain	24.56	19.71	885.64	1,231.89	15.96	13.07
	No Rain	27.65	20.03	986.57	1,355.76	16.28	14.15
Off-Peak Hour	Rain	28.7	20.25	756.34	1,001.55	16.73	13.28
	No Rain	30.5	21.68	500.68	899.56	17.54	15.02

Table 4 shows that when rain occurred during evening rush hour, passenger demand increased, whereas when rain occurred during off-peak hours, the demand declined. The passenger number in Kunming increased by approximately 24.6% during rainy evening rush hours compared with rush hours on rainless days. Nevertheless, the number declined by 9.22% during off peak hours. In Wuxi, the total number of passengers increased by approximately 10.72% during evening rush hours, while this number decreased by 9.21% during off-peak hours.

### The spatial analysis of taxi service

To gain a deeper insight into the spatial evolution of highly profitable passengers, we developed a convenient method to measure the demand for highly profitable taxi services. The computing process was fitted using the nearest-neighbour analysis and kriging method packages in ArcGIS version 10.0.

The detailed solution process is as follows. First, we determined the shape of the study area for Wuxi and Kunming based on their administrative region divisions. To facilitate comparisons between the cities, we identified rectangular areas that included both the city centres and suburbs and recorded the four latitude-longitude coordinates of the rectangular vertices. The distances corresponding to the rectangles’ widths and heights were calculated in the Mercator Projection based on the rules for coordinate transformations. The formulas used for the map-projection transform are as follows:

X=Lon⋅20037508.34/180 (2)

$$Y=Ln\left[\frac{\tan((90+Lat)\cdot \pi /360)}{\pi /180}\right]\cdot 20037508.34/180$$ (3)

where X and Y are the x-axis and y-axis coordinate values of the Mercator Projection, respectively; and Lat and Lon refer to the latitude and longitude coordinates of the four rectangular vertices, respectively. The difference between the x-axis or y-axis values of the corresponding vertices is the width or the height of a study area.

**Fig 5:**
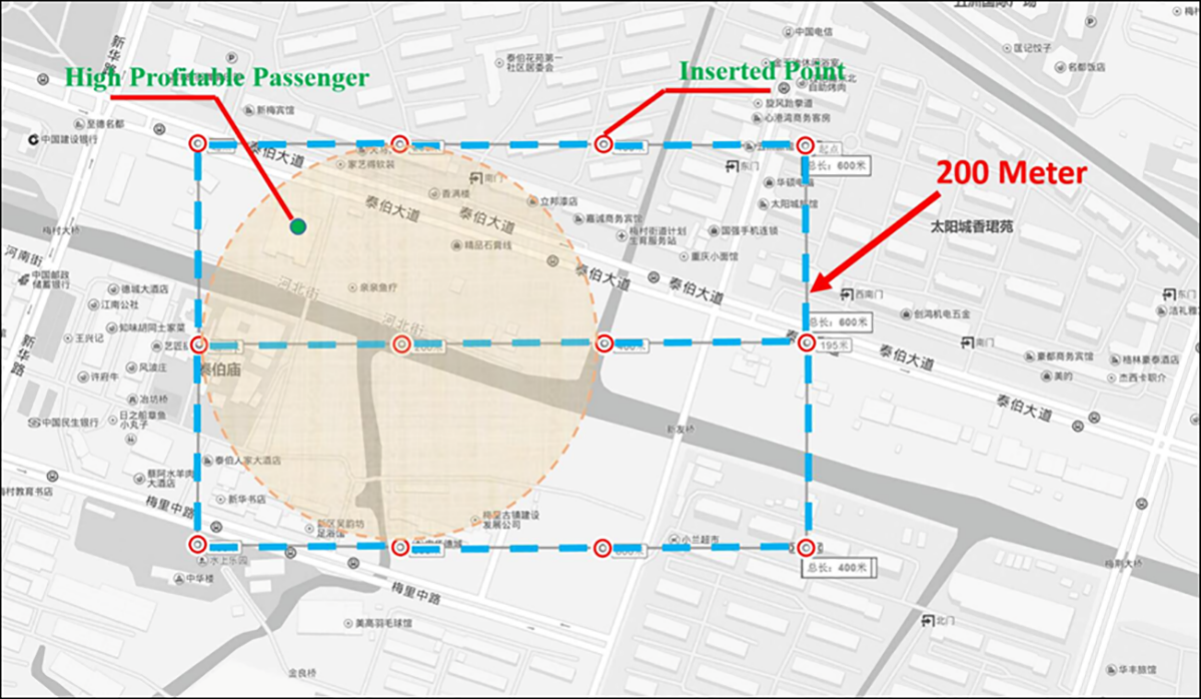
Distribution diagram for certain verification points.

Second, the verification points that cover the entire Wuxi and Kunming map layers were inserted. To measure the taxi service demand of unobservable regions, we applied an interpolation method to estimate the missing values that were caused by the image transformation process. In theory, a greater number of verification points on the map would correspond to better interpolation results. However, calculating a high number of verification values increased the complexity of the algorithm, which must be considered during image transformation. Thus, we treated the scope within a 200-meter radius as the fundamental measuring unit and used the Microsoft Visual Studio 5.0 integrated development environment (IDE) and the Visual Basic.NET language to insert a verification point every 200 metres (Fig 5). The total number of verification points for the Wuxi and Kunming map layers were 19,760 and 21,210, respectively.

Third, we assumed that the demand for highly profitable taxi service at each point was directly proportional to the value of the highly profitable passengers and inversely proportional to the Euclidean distance from there to the measuring unit's central position. The sum of the total rate for every highly profitable passenger covered within the scope was the total demand of that point’s taxi service. The calculation can be expressed as follows:

$$P_{i}=\sum_{k}\frac{E}{D}$$ (4)

where *P*_*i*_ is the demand of verification point *i*, *E* is defined the same as in Formula (1), and *D* is the Euclidean distance from the highly profitable passenger *k* to the scope's central position (as shown in Fig 5).

Finally, we applied the Kriging method from ArcGIS to complete the grid interpolation of the verification point layers for these three time intervals and transformed the resulting heat maps into a uniform measurement unit for the subsequent comparative analysis. Kriging interpolation is an unbiased optimal space-time estimation method for studying variables in a limited region based on variance function theory. This method considers the spatial positions of the interpolation points and the observed data points as well as the relationships between neighbouring points. Because this method utilizes the existing structural characteristics of the spatial distribution of the observed values, it is more accurate and more practical than traditional methods.

**Fig 6:**
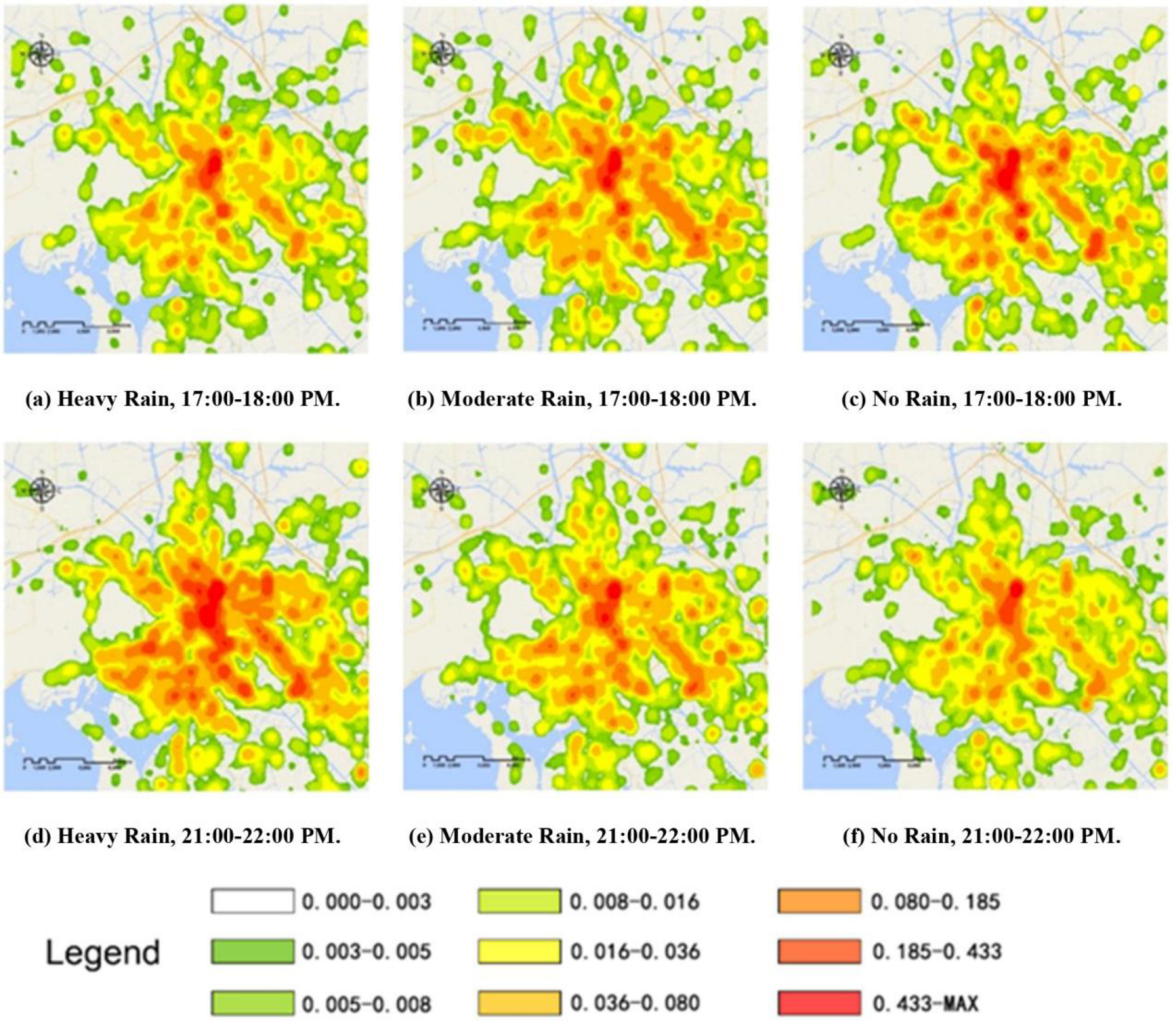
Spatial distribution of Wuxi taxi service demand.

**Fig 7:**
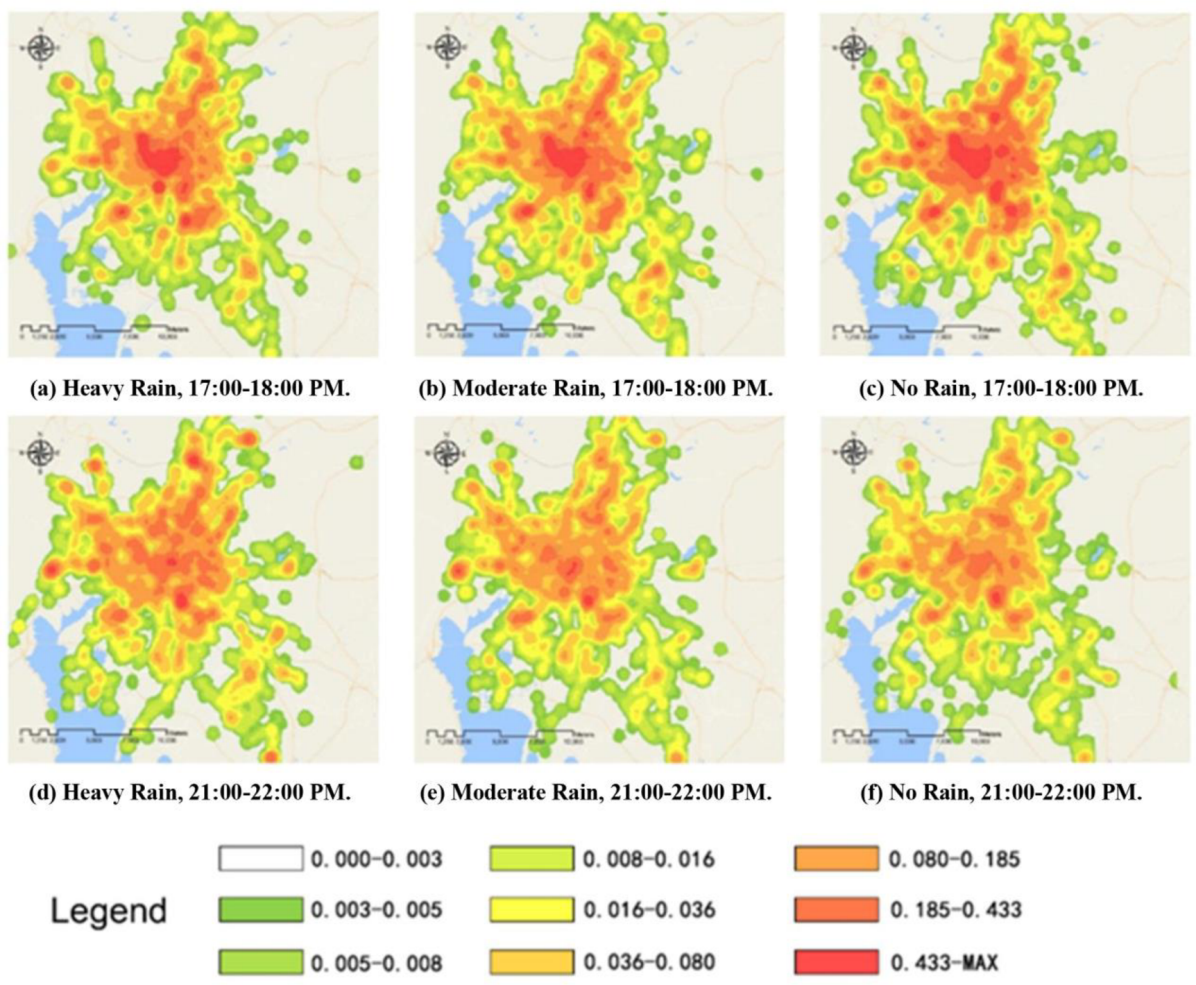
Spatial distribution of Kunming taxi service demand.

The spatial distributions of the Wuxi and Kunming taxi service demands for highly profitable passengers are shown in Figs 6 and 7, respectively.

From Figs 6 and 7 we can see that the spatial distribution of the demand for highly profitable taxi services is associated with land use characteristics. From the results of an earlier study[6], regardless of the weather, highly profitable passengers were primarily concentrated around large buildings in the central business districts (CBD) such as shopping centres, supermarkets, office buildings and transportation hubs. The demand for highly profitable taxi services weakened gradually from these regions to the suburban areas. To perform a comparative analysis of these three time intervals, the influences of rainfall on highly profitable passengers in Wuxi and Kunming are summarized in the following three points.

1. For the non-rush hour period (9:00 to 10:00 PM, Figs 6(A)–6(C) and 7(A)–7(C)), a large difference in highly profitable taxi service demand was observed between rainy and sunny days. According to the statistics of the verification points, under heavy and moderate rain conditions, taxi demand in Wuxi decreased by 8.03% and 6.45%, respectively, compared with the demand under sunny conditions. These findings were consistent with those in Kunming, where taxi demand under heavy and moderate rain conditions decreased by 11.13% and 4.22%, respectively, compared to sunny conditions. These results indicate that precipitation is the key influencing factor on the distribution and volatility of taxi service demand during non-rush hours. As the rainfall intensity increases, the taxi demand in the peripheral areas falls substantially, whereas the demand in the core central areas of the cities remains at a high level. This finding is related to the high activity-travel demand of residents in core areas regardless of the weather conditions. Moreover, the number of highly profitable passengers is directly proportional to the activity-travel demand, especially in good traffic environments.

2. During the evening rush hour period (5:00 to 6:00 PM, Figs 6(D)–6(F) and 7(D)–7(F)), a weak negative correlation was observed between the overall taxi demand and the number of highly profitable passengers (the top 25% of overall demand) under increases in the rainfall intensity. As shown in Table 3 and Table 4, the overall taxi service demand decreased by 8.03% in Wuxi and 11.13% in Kunming. However, the demand for highly profitable taxi service showed slight increases during precipitation because the number of people seeking taxi service increases under rainy conditions. A comparison of the results during non-rush hours showed that rainfall intensity had less effect on the absolute demand for taxi service (such as using a taxi service for commuting purposes). Moreover, on both rainy and sunny days, the best rush hour service strategy for taxi drivers to gain benefits is to avoid congested routes.

3. Excluding the central business districts, hospitals and middle schools, the areas with the fewest highly profitable passengers during non-rush hours on both sunny and rainy days included the recreational regions located at the borders between the older sections of the cities and the surrounding suburbs. In addition, compared to the spatial analysis of the non-rush hour period, the rush hour demand for highly profitable taxi services mainly involved the residential areas and traffic hubs at the outskirts of the cities. Therefore, on rainy days, taxi drivers' strategies should focus on the difference in demand between the rush hour and non-rush hour periods. Beyond encouraging taxi drivers to seek passengers in the CBD, the most effective approach is to guide them to increase their service time and opportunities in these corresponding regions.

## Conclusions

This paper investigated the potential impact of rainfall on taxi operations in the Chinese cities Wuxi and Kunming by analysing the spatio-temporal distributions of taxi service demands. To determine the relationship between precipitation and taxi driver operating efficiency, we used the concept of highly profitable passengers to calculate the demand for taxi services and applied two tools (nearest-neighbour analysis and the kriging method) from ArcGIS version 10.0 to visualize and explore the demand distribution. The results indicate that precipitation is a key influencing factor on the distribution and volatility of taxi service demand. As rainfall intensity increases, the taxi service demands for evening rush hour and non-rush hour periods on workdays show opposite trends. Therefore, taxi operating departments should guide cruising taxis to high demand regions based on the rainfall condition and the business hour.

Compared with previous studies, this study contributes to the research literature on taxi services in two main ways:

1. It verifies that the concept of highly profitable passengers meets the research requirement for viability and effectiveness in spatio-temporal evolution analysis of urban taxi services;
2. It demonstrates that the impact of rainy weather on taxi service demand, especially on highly profitable taxi service demand, is concentrated around the city central areas and that the most effective response for taxi drivers in Wuxi and Kunming to earn more money and limit discrepancies between taxi service demand and supply is to increase their service times.

However, two limitations with this study should be noted. The confidence in the results could be improved by analysing more extensive taxi GPS data and more informative weather record features. In addition, the methods and results presented in this research can be used as a practical reference for operating and dispatching urban taxi services. In future work, we plan to focus on obtaining more information using combinations of resources such as sensors, records from taxi-hailing apps, taxi drivers' financial records, and so on to build a simulation analysis that may improve the results.
